# Supplementary material for: Mechanisms governing the pioneering and redistribution capabilities of the non-classical pioneer PU.1
Source: Nat Commun. 2020 Jan 21;11:402. doi: 10.1038/s41467-019-13960-2 (PMC6972792; doi:10.1038/s41467-019-13960-2)
Supplement: Supplementary file 7 — Source data [file 41467_2019_13960_MOESM7_ESM.zip › Source_Data/Figure6/Figure6B_MotifScanOutput/FLI1_PU.1/homerResults/motif27.similar.html]

motif27

## Information for motif27

C
T
A
G
A
C
T
G
A
C
T
G
A
G
T
C
A
C
T
G
A
C
T
G
C
T
A
G
C
T
A
G
A
G
T
C
  
Reverse Opposite:  

A
C
T
G
G
A
T
C
G
A
T
C
G
T
A
C
A
G
T
C
C
T
A
G
A
G
T
C
A
G
T
C
G
A
T
C
  

|  |  |
| --- | --- |
| p-value: | 1e-51 |
| log p-value: | -1.177e+02 |
| Information Content per bp: | 1.807 |
| Number of Target Sequences with motif | 738.0 |
| Percentage of Target Sequences with motif | 7.42% |
| Number of Background Sequences with motif | 1338.1 |
| Percentage of Background Sequences with motif | 3.67% |
| Average Position of motif in Targets | 102.1 +/- 56.6bp |
| Average Position of motif in Background | 94.3 +/- 67.1bp |
| Strand Bias (log2 ratio + to - strand density) | -0.1 |
| Multiplicity (# of sites on avg that occur together) | 1.20 |
| Motif File: | file (matrix) reverse opposite |

### Similar de novo motifs found

|  |  |  |  |  |  |  |  |
| --- | --- | --- | --- | --- | --- | --- | --- |
| Rank | Match Score | Redundant Motif | P-value | log P-value | % of Targets | % of Background | Motif file |
| 1 | 0.925 | C T A G A T C G A T C G G A T C C A T G C A T G C T A G C A T G A G T C A G T C C G A T T G C A | 1e-47 | -109.087137 | 5.89% | 2.71% | motif file (matrix) |
| 2 | 0.944 | C T A G A C T G A C T G A T G C A C T G A C T G C T A G A C T G A G T C G A T C G C T A | 1e-45 | -104.113913 | 4.21% | 1.66% | motif file (matrix) |
